# Supplementary material for: Osteogenic growth peptide is a potent anti-inflammatory and bone preserving hormone via cannabinoid receptor type 2
Source: eLife. 2022 May 23;11:e65834. doi: 10.7554/eLife.65834 (PMC9154745; doi:10.7554/eLife.65834)
Supplement: Supplementary file 1. — The 10 agonists that are also endogenous proteins/peptides are highlighted in bold fonts. PTX, pertussis toxin. [file elife-65834-supp1.docx]

| **Name** | **Known Gi or unknown GPCR** | **Effect (agonist/antagonist)** | **Targeted cells** | **Molecule type (protein, peptide, lipid, other)** | **Source (PMID)** |
| --- | --- | --- | --- | --- | --- |
| Phospholipids lysophosphatidic acid (LPA) | LPA1 (Gi) | Gi-dependent proliferative and/or anti-apoptotic activities | Osteoblasts | Lipid | 27643449, 11181524 |
| Sphingosine-1-phosphate (S1P) | S1PR1, S1PR2, S1PR3 (Gi) | Gi-dependent proliferative and/or anti-apoptotic activities and inhibits adipogenesis | Osteoblasts | Lipid | 25445169 |
| AEA and 2AG | CB1 (Gs/Gi), CB2 (Gi) | Osteoblast proliferation and osteoclast inhibition | Osteoclasts and osteoblasts | Lipid | 20803555 |
| Oleoyl Serine | Gi (inhibited by PTX) | Osteoblast proliferation, osteoclastogenesis inhibition | Osteoclasts and osteoblasts | Lipid | 20876113 |
| Fluoroaluminate | Gi (inhibited by PTX) | Proliferative | Osteoblasts | Mineral | 9797473 |
| Nor-epinephrine, Epinephrine | α1β adenoR coupling to a Gi GPCR | Agonist of α-adrenergic receptor via coupling to a Gi GPCR and stimulates osteoblast proliferation | Osteoblasts | Catecholamine | 23061915, 11425646, 10385412, 9737341 |
| biphenyl | CB2 | Non endogenous inverse agonist | Osteoclasts and osteoblasts | aromatic hydrocarbon | 21867920 |
| dihydroxycholesterol / Oxyterol | EBI2 Gi | Reduces osteoclast precursor migration in vivo. Defective EBI2 signaling led to increased bone mass in male mice and protected female mice from age- and estrogen deficiency–induced osteoporosis. | Osteoclasts | Lipid | 26511861, 26438360 |
| Gadolinium (Gd) | Orphan GPCR (Gi) | Activates a Gi to suppress the MAPK pathway in PC3, suppresses osteoclast differentiation *in vitro* | Osteoclasts | Natural element | 21605080 |
| lanthanum (La3+ ionized form of lanthanum) | Orphan GPCR (Gi) | Enhances *in vitro* osteoblast differentiation via PTX-sensitive Gi protein and ERK signaling pathway | Osteoblasts | Natural element | 18810761 |
| Leukotriene B4 | Gi, BLT1/2 | Leukotriene B4 increases the osteoclast activity through the BLT1-Gi protein-Rac1 signaling pathway | Osteoclasts and osteoblasts | Endogenous inflammatory chemicals | 19965376 |
| PGE1 (and other PGs) | Gi (EP3 receptor) | PGE1 in Ob binds EP3 (Gi) but also EP2 and EP4 which are Gs GPCRs. Stimulates osteogenic activity. | Osteoblasts | Lipid | 15062850 |
| ADP | P2Y13 | P2Y13 deficiency is associated with decreased bone mass but increased osteogenic response to loading | Osteoblasts | Nucleotide | 12376532 |
| **Apelin** | APJ | Proliferative and anti-apoptosis but complete KO induces high bone mass phenotype | Osteoblasts | **Protein** | 16563531, 23584856 |
| **Chemokines** | CXCR4 (Gi) and other CX receptors (mostly Gq) | Adverse effects depending on the receptor | Osteoclasts | **Protein** | 24408534 |
| **Serum Amyloid A3 (Saa3)** | Fpr2 (Gi) | Secreted by osteoclasts to block the cAMP increase in osteoblasts (by PTH) - inhibits PTH-stimulated osteoblastic differentiation | Osteoblasts | **Protein** | 26703472 |
| **Osteogenic growth peptide** | Unknown Gi | Mitogenic in osteoblasts | osteoblasts | **Protein** | 11329614 |
| **CXCL10** | Unknown Gi | Contributes to bone loss | T-cells | **Protein** | 21708014 |
| **CNP** | Unknown Gi | Stimulates osteoblast differentiation but attenuates proliferation | Osteoblasts | **Peptide** | 17562543 |
| Adenosine | A3-adenosine receptor Gi | Prevents bone resorption | Osteoclasts | Nucleotide | 16956430 |
| **Degradation products of Secreted phosphoprotein (Spp) 24 kDa** | Gi | Inhibition of osteoblasts differentiation | Osteoblasts | **Peptides** | 25501958 |
| **Melatonin** | MT2 (Gi) | Positive regulation on bone mass, promotes osteoblast differentiation and bone formation | Osteoblasts | **Protein** | 18507714  15303021  10419530 |
| **Epidermal growth factor** | Gi | Bone anabolic | Osteoblasts | **Protein** | 9797479 |
| **FSH** | FSHR (Gi) | Increases osteoclastogenesis | Osteoclasts | **Polypeptide** | 20171951  17681281 |
