# Supplementary material for: Osteogenic growth peptide is a potent anti-inflammatory and bone preserving hormone via cannabinoid receptor type 2
Source: eLife. 2022 May 23;11:e65834. doi: 10.7554/eLife.65834 (PMC9154745; doi:10.7554/eLife.65834)
Supplement: Supplementary file 2. [file elife-65834-supp2.docx]

| **Binding complex** | **Inactive CB2_X-ray_** | **Active CB2_HM_** |
| --- | --- | --- |
| OGP in ECL | -7.71 | -9.29 |
| OGP in TM | -10.59 | -11.62 |
| OGP in ECL in the presence of CP55,940 in TM | -7.09 | -9.35 |
