# Supplementary material for: Osteogenic growth peptide is a potent anti-inflammatory and bone preserving hormone via cannabinoid receptor type 2
Source: eLife. 2022 May 23;11:e65834. doi: 10.7554/eLife.65834 (PMC9154745; doi:10.7554/eLife.65834)
Supplement: Supplementary file 3. [file elife-65834-supp3.docx]

| **Region** | **CB1** | **CB2** |  |  | CB1-CB2 homology | | |
| --- | --- | --- | --- | --- | --- | --- | --- |
|  |  |  | **#AA in CB1** | **#AA in CB2** | **#Identical AA** | **%I** | %S |
| Full sequence | 1-472 | 1-360 | 472 | 360 | 145 | **30** | 43 |
| ECL1+2+3 |  |  | 24 | 22 | 9 | **38** | 50 |
| TM1-7 |  |  | 233 | 234 | 120 | **51** | 69 |
| N-Ter | 1-111 | 1-28 | 111 | 28 | 4 | **4** | 9 |
| TM1 | 112-144 | 29-61 | 33 | 33 | 15 | **45** | 64 |
| ICL1 | 145-149 | 62 -66 | 5 | 5 | 2 | **40** | 40 |
| TM2 | 150-179 | 67-96 | 30 | 30 | 20 | **67** | 80 |
| ECL1 | 180-184 | 97-101 | 5 | 5 | 3 | **60** | 60 |
| TM3 | 185-220 | 102-137 | 36 | 36 | 23 | **64** | 78 |
| ICL2 | 221-228 | 138-145 | 8 | 8 | 3 | **38** | 75 |
| TM4 | 229-254 | 146-171 | 26 | 26 | 10 | **38** | 73 |
| ECL2 | 255-271 | 172-186 | 17 | 15 | 6 | **35** | 47 |
| TM5 | 272-312 | 187-225 | 41 | 39 | 13 | **32** | 49 |
| ICL3 | 313-331 | 226-230 | 19 | 5 | 1 | **5** | 11 |
| TM6 | 332-369 | 231-271 | 38 | 41 | 19 | **46** | 68 |
| ECL3 | 370-371 | 272-273 | 2 | 2 | 0 | **0** | 50 |
| TM7 | 372-400 | 274-302 | 29 | 29 | 20 | **69** | 83 |
| H8 | 401-413 | 303-319 | 13 | 17 | 2 | **12** | 29 |
| C-Ter | 414-472 | 320-360 | 59 | 41 | 4 | **7** | 12 |
