# Supplementary material for: Osteogenic growth peptide is a potent anti-inflammatory and bone preserving hormone via cannabinoid receptor type 2
Source: eLife. 2022 May 23;11:e65834. doi: 10.7554/eLife.65834 (PMC9154745; doi:10.7554/eLife.65834)
Supplement: Transparent reporting form [file elife-65834-transrepform1.docx]

***eLife’s* transparent reporting form**

We encourage authors to provide detailed information *within their submission* to facilitate the interpretation and replication of experiments. Authors can upload supporting documentation to indicate the use of appropriate reporting guidelines for health-related research (see [EQUATOR Network](http://www.equator-network.org/%20)), life science research (see the [BioSharing Information Resource](https://biosharing.org/)), or the [ARRIVE guidelines](http://www.plosbiology.org/article/info:doi/10.1371/journal.pbio.1000412) for reporting work involving animal research. Where applicable, authors should refer to any relevant reporting standards documents in this form.

If you have any questions, please consult our Journal Policies and/or contact us: [editorial@elifesciences.org](mailto:editorial@elifesciences.org).

**Sample-size estimation**

- You should state whether an appropriate sample size was computed when the study was being designed
- You should state the statistical method of sample size computation and any required assumptions
- If no explicit power analysis was used, you should describe how you decided what sample (replicate) size (number) to use

Please outline where this information can be found within the submission (e.g., sections or figure legends), or explain why this information doesn’t apply to your submission:

All details related to sample size and statistical methods for every experiment are indicated in the end of each legend of each figure.

The appropriate sample size was decided when the study was being designed.

The appropriate statistical tests for every experiment and the correct methods were used to decipher the results.

We decided the sample size based on previous published experiments.

**Replicates**

- You should report how often each experiment was performed
- You should include a definition of biological versus technical replication
- The data obtained should be provided and sufficient information should be provided to indicate the number of independent biological and/or technical replicates
- If you encountered any outliers, you should describe how these were handled
- Criteria for exclusion/inclusion of data should be clearly stated
- High-throughput sequence data should be uploaded before submission, with a private link for reviewers provided (these are available from both GEO and ArrayExpress)

Please outline where this information can be found within the submission (e.g., sections or figure legends), or explain why this information doesn’t apply to your submission:

Every experiment was performed at least 3 times in the period of 5 years and results were verified in two different laboratories. In the case of in vivo experiments, the definition of biological replication is the number of animals in every treatment and the technical replication is the number of measurements taken from each animal, the mean of 3 measurements was calculated from each animal in the same treatment. In the case of in vitro experiments, the definition of biological replication is the treatment sample size and the technical replication is done in triplicates for each biological replicate. No data was excluded from the final results. Some outliers were excluded from the results, in vitro outliers were excluded when contamination was detected and in vivo outliers were excluded when technical errors were detected. Biological and technical replicates performed for each experiment is described in the legend of every figure.

**Statistical reporting**

- Statistical analysis methods should be described and justified
- Raw data should be presented in figures whenever informative to do so (typically when N per group is less than 10)
- For each experiment, you should identify the statistical tests used, exact values of N, definitions of center, methods of multiple test correction, and dispersion and precision measures (e.g., mean, median, SD, SEM, confidence intervals; and, for the major substantive results, a measure of effect size (e.g., Pearson's r, Cohen's d)
- Report exact p-values wherever possible alongside the summary statistics and 95% confidence intervals. These should be reported for all key questions and not only when the p-value is less than 0.05.

Please outline where this information can be found within the submission (e.g., sections or figure legends), or explain why this information doesn’t apply to your submission:

As described in the "methods and materials" section, for a sample size greater than or equal to 6 and with normal distribution, differences between time/treatment groups were analyzed by Student’s t-test (for 2 groups only) or ANOVA (for 3 groups or more). When significant differences were indicated by ANOVA, the group means were compared using the Fisher-LSD test for pairwise comparisons. Multiple group comparisons with n<6 were analyzed using non-parametric ANOVA. A p value less than 0.05 was considered statistically significant. For the human serum analysis, we used both the Kruskal-Willis one-way ANOVA and the Mann Whitney-Wilcoxon rank sum tests. The N per group is indicated for every experiment in the legend of the figure. All results are described with the appropriate SD.

(For large datasets, or papers with a very large number of statistical tests, you may upload a single table file with tests, Ns, etc., with reference to sections in the manuscript.)

**Group allocation**

- Indicate how samples were allocated into experimental groups (in the case of clinical studies, please specify allocation to treatment method); if randomization was used, please also state if restricted randomization was applied
- Indicate if masking was used during group allocation, data collection and/or data analysis

Please outline where this information can be found within the submission (e.g., sections or figure legends), or explain why this information doesn’t apply to your submission:

No masking was used during group allocation and data collection. Animals for the experimental groups were chosen randomly and no restricted randomization was applied. Human samples were collected from humans from the appropriate age and gender as described in the "methods and materials" section.

**Additional data files (“source data”)**

- We encourage you to upload relevant additional data files, such as numerical data that are represented as a graph in a figure, or as a summary table
- Where provided, these should be in the most useful format, and they can be uploaded as “Source data” files linked to a main figure or table
- Include model definition files including the full list of parameters used
- Include code used for data analysis (e.g., R, MatLab)
- Avoid stating that data files are “available upon request”

Please indicate the figures or tables for which source data files have been provided:

Data was calculated via GraphPad Prism 8 software.

All source data files are attached.
